# A Rare Case of Hemichorea-Hemiballismus Due to Chronic Uncontrolled Hyperglycemia

**DOI:** 10.7759/cureus.10861

**Published:** 2020-10-09

**Authors:** Qalb A Khan, Anisa Batool, Muhammad Adnan Haider, Muhammad Hanif, Abdul Wali Khan

**Affiliations:** 1 Internal Medicine, Crozer Chester Medical Center, Chester, USA; 2 Internal Medicine, Jinnah Hospital Lahore, Pakistan, PAK; 3 Internal Medicine, Allama Iqbal Medical College/Jinnah Hospital, Lahore, PAK; 4 Internal Medicine, Khyber Medical College, Hayatabad Medical Complex, Peshawar, PAK; 5 Internal Medicine, College of Physician and Surgeons Pakistan, Peshawar, PAK; 6 Internal Medicine, Hayatabad Medical Complex, Peshawar, PAK

**Keywords:** hemichorea-hemiballismus, uncontrolled hyperglycemia

## Abstract

Chronic uncontrolled hyperglycemia is a rare yet important cause of hemichorea-hemiballismus and very common among postmenopausal women. This case report illustrates the importance of recognizing hyperglycemia as a potential cause of hemiballismus. There is a need to differentiate hyperglycaemic intracranial changes from other intracranial pathologies, as prompt glycemic control can help improve hemiballistic symptoms and prevent a more aggressive or invasive workup.

## Introduction

In 1960, the association between hemichorea and non-ketotic hyperglycaemic (NKH) was described for the first time. The possible association between non-ketotic hyperglycemia and focal neurological deficits, such as choreoathetosis and ballismus, was first delineated in 1960. Surprisingly, this association is more prevalent in Asian women suggesting some genetic components might be involved in such association [1-2]. Hemiballism is characterized by irregular, coarse, fierce, flinging movements of the limbs particularly due to the contraction of proximal muscles while chorea presents as continuous, random, jerking movements involving both proximal and distal muscles [3]. Diabetic striatopathy, defined as chorea/ballismus, which is associated with the hyperglycaemic condition and peculiar reversible abnormality of basal ganglia on computed tomography (CT) and/or magnetic resonance imaging (MRI) [4] is a rarely diagnosed complication of diabetes and although it is more often associated with type 2 diabetes but can also be seen in patients with type 1 diabetes [5]. We present a case of NKH-associated hemiballismus/hemichorea that progressed to involve all extremities resulting in chorea/ballismus to underpin the importance of prompt diagnosing and treating the underlying hyperglycemia and subsequent prevention of aggressive or invasive workup.

## Case presentation

**Figure 1:**
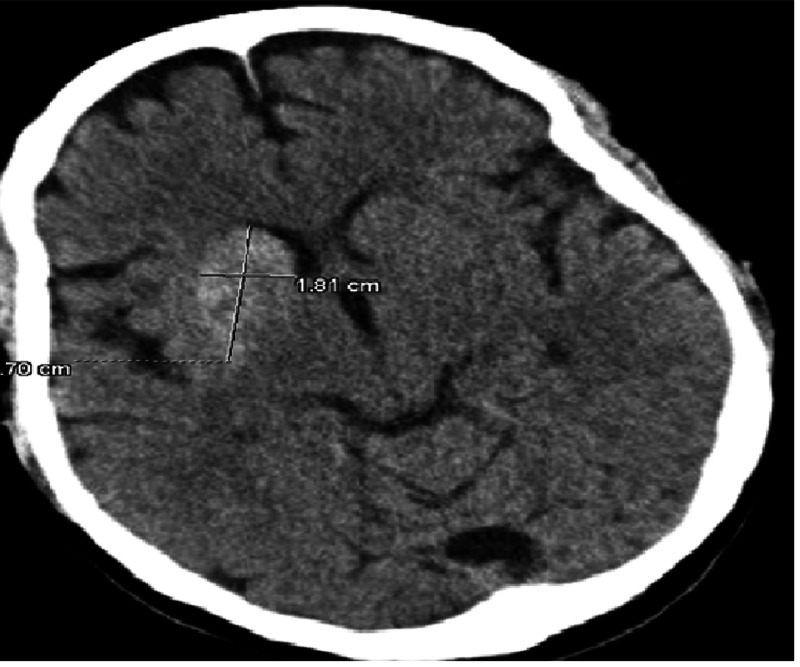
CT head showing hyperdensity (probable hemorrhage) in the right caudate nucleus head and anterior putamen CT: computed tomography

A 48-year-old Caucasian female with a past medical history of uncontrolled type II diabetes mellitus presented with ballistic movements of the left upper extremity (LUE), which progressed to the whole body within three days. There were no other neurological deficits. No abnormality was seen on neurological examination except uncontrolled body movements. Initial labs were normal except hyperglycemia (600 mg/dL) and hyponatremia. Her initial CT head showed an area of a 3.7 x 1.8 cm hemorrhage surrounding a 2.1 x 1.5 cm probable infarct in the right caudate nucleus head and anterior putamen, with the intervening anterior limb of the internal capsule (Figure 1). However, an infectious etiology needed to be ruled out as well. Stat MRI was recommended by a radiologist.

**Figure 2:**
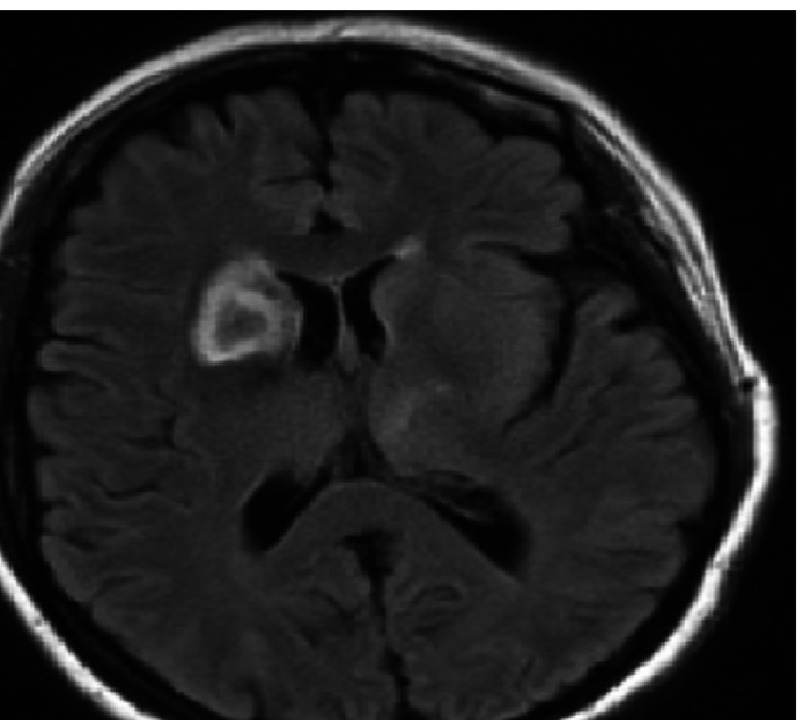
MRI brain showing hyperattenuation in the right caudate nucleus head and right lentiform nucleus An area of hypoattenuation can be also seen in the caudate nucleus head. MRI: magnetic resonance imaging

MRI showed a lesion in the right area of the brain and the differential diagnosis included infectious or inflammatory changes versus an underlying primary glial neoplasm or hemorrhage (Figure 2). Correlation with cerebrospinal fluid (CSF) was suggested for further characterization.

**Figure 3:**
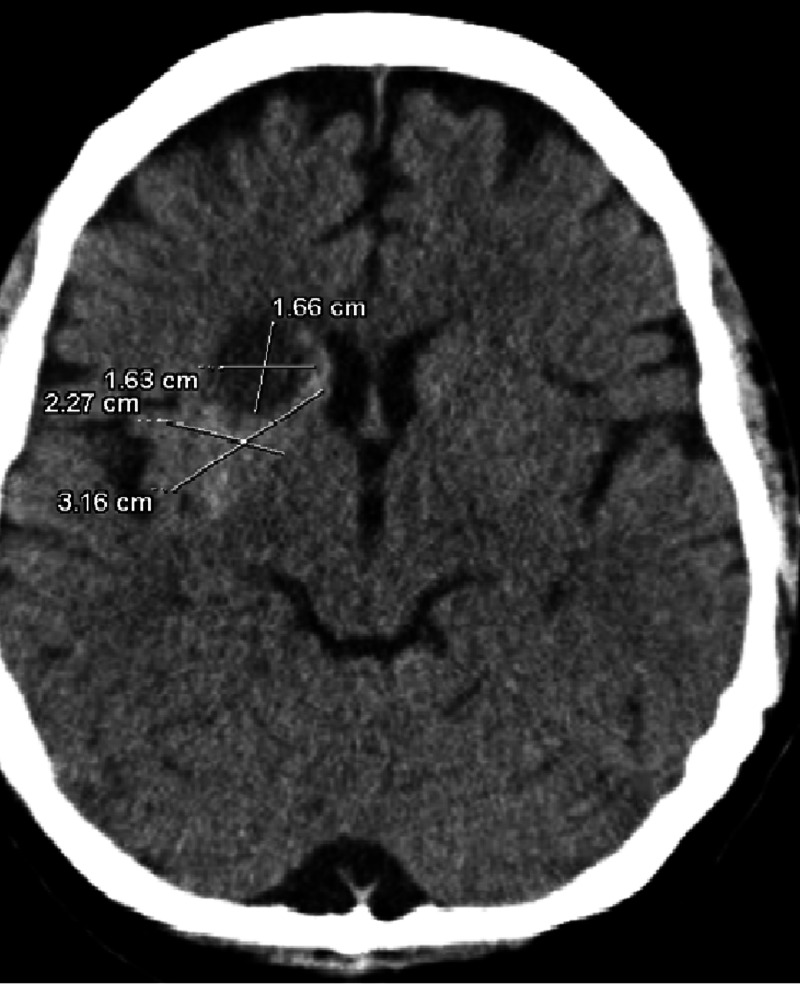
CT head shows hyperdense right caudate nucleus head and lentiform nucleus suggesting osmotic myelinolysis secondary to chronic uncontrolled non-ketotic hyperglycemia CT: computed tomography

Meanwhile, the patient became very combative and restless, lost IV access. An intraosseous infusion was placed, the patient was intubated and a lumbar puncture (LP) was done. Additionally, the patient was started on broad-spectrum antibiotics. The patient’s cerebrospinal fluid (CSF) analysis returned within normal limits. The patient was evaluated by the neurology and neurosurgery department and their differential diagnosis included glioblastoma/hemorrhage/infectious etiology or subacute ischemic stroke. A follow-up CT head was recommended. CT showed a focal area of hypoattenuation in the right caudate nucleus head, anterior limb right internal capsule, and anterior right lentiform nucleus representing osmotic myelinolysis secondary to chronic uncontrolled non-ketotic hyperglycemia (Figure 3).

Given the history of uncontrolled hyperglycemia and CT findings, she was diagnosed with NKH-associated hemiballismus/hemichorea. The patient stayed intubated for about a week or so, as she developed aspiration pneumonia. Her blood glucose was strictly controlled. Once extubated, the patient’s involuntary movements improved. Of note, the patient’s glycated hemoglobin (HbA1c) was in the range of 13 to 17 over the last year.

## Discussion

Hemiballism is characterized by irregular, coarse, violent, flinging movements of the limbs, particularly due to the contracting of proximal muscles while chorea presents as continuous, random, jerking movements involving both distal and proximal muscles [3]. The two most common etiologies of hemiballism are stroke (ischemic or hemorrhagic) and non-ketotic hyperglycemia. Other causes, including encephalitis, traumatic brain injury, autoimmune problems, cerebral toxoplasmosis associated with acquired immunodeficiency syndrome (AIDS), mass lesions (neoplasm/cysts), multiple sclerosis, drugs (levodopa, oral contraceptives, and anticonvulsants), metabolic derangements in levels of sodium, calcium, magnesium, and manganese; uremia, toxins such as carbon monoxide, alcohol, aluminum, and lead; and Wilson disease, have been reported [6-7]. These abnormal dyskinetic movements are associated with a subthalamic lesion - a lens-shaped group of nuclei, which inhibits the activity of the ventrolateral thalamus through increased secretion of gamma-aminobutyric acid (GABA) from globus pallidus [8]. The underlying pathophysiology of chorea or ballismus associated with non-ketotic hyperglycemia is poorly understood. However, the following mechanisms are proposed [9]: (a) hyperglycemia and associated hyperviscosity disrupt the blood-brain barrier, leading to acidosis and regional metabolic failure; (b) striate infarction or petechial hemorrhages; (c) increased sensitivity of post-menopausal dopamine receptors can trigger hyperkinetic movements; (d) utilization of gamma-aminobutyric acid (GABA) during non-ketotic hyperglycemic episodes depletes it in thalami/striatum. Through the use of integrated positron emission tomography (PET) and CT scans, a profound decrease in glucose metabolism, particularly in the contralateral striatum, has been demonstrated as the possible mechanism [10].

Almost all the patients who present with non-ketotic hyperglycemic-associated hemichorea/hemiballismus are found to have high blood glucose levels and hyperintense striatum on MRI. CT head and MRI brain of these patients show striatal (putamen) hyperdensity and hyperintensity respectively [11-12]. Our patient presented with typical symptoms of non-ketotic hyperglycaemic associated hemichorea/hemiballismus, and CT head suggested hemorrhage. She was suspected to have a stroke; a stat MRI was ordered and findings were suggestive of infectious or inflammatory changes versus glial neoplasm or hemorrhage. Based on the MRI findings, lumbar puncture (LP) was done under general anesthesia, as the patient was non-cooperative and combative. CSF analysis was within normal limits. Meanwhile, she was diagnosed with non-ketotic hyperglycaemic-associated hemichorea/hemiballismus based on the findings of repeat CT head and history. However, she developed ventilator-associated pneumonia. This case is important to make physicians cognizant of the presenting symptoms of non-ketotic hyperglycaemic-associated hemichorea/hemiballismus and the subsequent avoiding of aggressive and invasive workup while they encounter a person with diabetes presenting with hyperkinetic disorders because the symptoms usually resolve after the correction of glucose derangements. However, sometimes, dopamine depletion agents, such as haloperidol or tetrabenazine [13], and rarely for refractory cases, repetitive transcranial magnetic stimulation [14] or deep brain stimulation [15] are needed to control the symptoms. Symptoms usually resolve completely within six months to one year but, sometimes, chorea can persist for one to two years [16].

## Conclusions

In our case, hemiballismus and hemichorea were found in association with non-ketotic hyperglycemia. The physicians should keep in the mind this possible cause of hemiballismus or hemichorea while encountering a diabetic patient because early diagnosis can prevent unnecessary and invasive interventions.
